# Detection of lisdexamfetamine and its metabolite *d*-amphetamine in urine and gastric contents collected from a cadaver at forensic autopsy

**DOI:** 10.1007/s11419-022-00654-6

**Published:** 2022-12-23

**Authors:** Suguru Torimitsu, Kanju Saka, Kanako Noritake, Akira Namera, Yohsuke Makino, Rutsuko Yamaguchi, Hirotaro Iwase

**Affiliations:** 1 Department of Forensic Medicine, Graduate School of Medicine, The University of Tokyo, 7-3-1 Hongo,, Bunkyo-Ku, Tokyo 113-0033 Japan; 2 Department of Legal Medicine, Graduate School of Medicine, Chiba University, 1-8-1 Inohana, Chuo-Ku,, Chiba-Shi, Chiba 260-8670 Japan; 3 Department of Forensic Medicine, Graduate School of Biomedical and Health Sciences, Hiroshima University, Kasumi 1-2-3, Minami-Ku, Hiroshima, 734-8553 Japan

**Keywords:** Lisdexamfetamine (LDX), Amphetamine, Autopsy samples, Optical isomer separation, Stability of LDX in whole blood, LC–MS/MS

## Abstract

**Purpose:**

Lisdexamfetamine (LDX), which is used for the treatment of attention-deficit/hyperactivity disorder and narcolepsy, is composed of l-lysine attached to dextroamphetamine (*d*-amphetamine). In this article, we report a forensic autopsy case in which prescription drugs were unknown at autopsy. While amphetamine was detected, methamphetamine could not be detected by liquid chromatography–tandem mass spectrometry (LC–MS/MS) in any of samples collected. Thus, we aimed to quantify LDX concentrations in autopsy samples and to prove that the amphetamine detected in this case was due to metabolized LDX.

**Methods:**

Femoral vein blood, cardiac whole blood, urine, and gastric content samples were taken at autopsy for toxicological analysis. Qualitative and quantitative analyses were performed using LC–MS/MS. In addition, optical isomer separation for the amphetamine detected was conducted. The stability of LDX in whole blood and urine was also examined at three different temperatures.

**Results:**

The concentrations of LDX were < 4.00, 30.9, and 4.42 ng/mL in whole blood, urine, and gastric content samples, respectively. The concentrations of amphetamine were 329, 510, 2970, and 915 ng/mL in femoral vein blood, heart whole blood, urine, and gastric contents, respectively. The amphetamine detected in this case was identified to be only *d*-amphetamine by optical isomer separation. The *d*-amphetamine detected was considered to be derived from LDX. Stability experiments revealed that LDX in whole blood decreased at ambient temperature.

**Conclusions:**

The results in the present case report may be useful in interpreting whether or not the amphetamine detected in a cadaver is a metabolite of LDX.

**Supplementary Information:**

The online version contains supplementary material available at 10.1007/s11419-022-00654-6.

## Introduction

**Fig. 1:**
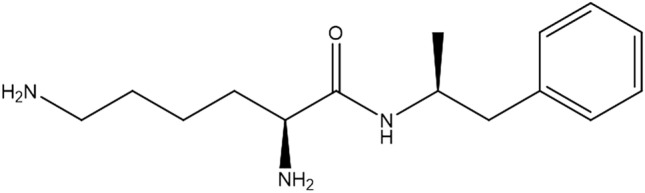
Structure of lisdexamfetamine (LDX)

Amphetamine is a sympathomimetic amine that increases brain levels of norepinephrine, serotonin, and dopamine by promoting the release and inhibiting the reuptake of these neurotransmitters [1, 2]. Typical acute effects after amphetamine use include enhanced attention and alertness, increased psychomotor performance, and loss of appetite [3]. Excessive amphetamine use can cause serious intoxication and death, predominantly by acute cardiac or cardiopulmonary failure, cerebrovascular hemorrhage, or hyperthermia [4–6]. Amphetamine is still one of the most frequently abused drugs worldwide [7] and is frequently detected in forensic toxicological cases [8]. On the other hand, amphetamine is used for the treatment of medical conditions such as attention-deficit/hyperactivity disorder (ADHD) and narcolepsy [9]. In 2007, lisdexamfetamine (LDX) (Vyvanse®) (Fig. 1) was approved for ADHD medication in the United States. LDX was also approved in Japan in 2019. LDX is composed of the amino acid l-lysine attached to dextroamphetamine (*d*-amphetamine) [10]. After oral ingestion, LDX is hydrolyzed primarily in the red blood cells to produce *d*-amphetamine [10, 11]. Therefore, when LDX is taken internally, amphetamine is predominantly detected in blood and urine.

In Japan, although methamphetamine is commonly abused as a recreational drug [12], abuse of amphetamine is considerably rare. Therefore, amphetamine is rarely detected alone in drug screening and is usually detected with precursor methamphetamine. In this report, we describe a forensic autopsy case in which prescription drugs were unknown at the stage of autopsy; although amphetamine was detected in qualitative screening analysis using liquid chromatography–tandem mass spectrometry (LC–MS/MS), methamphetamine was not found. Based on the later information from the police, the possibility of LDX administration arose for accounting for the appearance of amphetamine only.

## Case history and autopsy findings

A man in his twenties with a history of ADHD, depression, and panic attacks sent a suicide-like message to his mother. Half an hour later, an explosion sound was heard near his house, and a police officer discovered a fire in his room. Firefighters found him lying in a supine position with cardiopulmonary arrest inside the front door. Cardiopulmonary resuscitation was not performed. Three 4-L cans of white gasoline were found on the balcony, and oily components were detected in two of the cans. The site of ignition was determined to be inside his room. Although the police ruled it suicidal, the possibility of arson by another person was not excluded. Before the autopsy, the police did not investigate the hospital where he had been under medical treatment.

An autopsy was performed about 41 h after the death was confirmed. His length and weight were approximately 165 cm and 57.2 kg, respectively. External examinations demonstrated that most of the skin was brownish to blackish brown with scattered charring. Erythema was present on the posterior lower half of the right lower extremity. On the chest and the anterior surface of the upper limbs, there were skin lacerations exposing subcutaneous tissue, muscle, and some bones. Conjunctival petechiae were not found. Internal examinations showed a small amount of black soot in the upper trachea and reddening in the trachea and bronchial mucosa. Femoral vein blood, cardiac whole blood, urine, and gastric content samples were collected for drug examination.

## Materials and methods

### Reagents and materials

LDX dimesylate, *d*-amphetamine sulfate, and *l*-amphetamine sulfate were provided by the Japanese Ministry of Health, Labour and Welfare. Venlafaxine hydrochloride, *O*-desmethylvenlafaxine, and phentermine (internal standard, IS) were purchased from Tokyo Chemical Industry Co., Ltd. (Tokyo, Japan); venlafaxine-*d*_6_ from Toronto Research Chemicals, Inc. (Toronto, Canada); bromazepam, methanol (LC/MS grade), and acetonitrile (LC/MS grade) from FUJIFILM Wako Pure Chemical Corporation (Osaka, Japan); bromazepam-*d*_4_ methanol solution (100 µg/mL) and β-glucuronidase (*Helix pomatia*, type HP-2, ≥ 100,000 units/mL) from Merck (Darmstadt, Germany). All reagents were of analytical grade. Human whole blood and urine (used as blank matrices) were purchased from BioIVT (London, UK); the Micro Volume QuEChERS Kit™ from Shimadzu Corporation (Kyoto, Japan); and cryogenic vials (4 mL) from Sunitomo Bakelite Co., Ltd. (Tokyo, Japan). Ultrapure water was prepared using an Arium^®^ II purification system (Sartorius, Goettingen, Germany).

Stock standard solutions of LDX, *d*- and *l*-amphetamine, venlafaxine, *O*-desmethylvenlafaxine, bromazepam, phentermine, and venlafaxine-*d*_6_ were prepared in methanol (1 mg/mL) and stored at − 20 °C. Working solutions were prepared by appropriately diluting the stock solutions with acetonitrile.

An IS mixture solution was prepared by diluting the stock solutions of phentermine, venlafaxine-*d*_6_, and bromazepam-*d*_4_ to a concentration of 5000 ng/mL each. Working solutions were stored at 4 °C.

### Basic toxicological analyses

Ethanol and cyanide in blood and urine were examined by headspace gas chromatography (GC)–MS/nitrogen-phosphorus detection [13]. Drug screening was performed using the DRIVEN-FLOW^®^ M8-Z (Alfa Scientific Designs, Inc., Poway, CA, USA) device for immunoassays and LC–MS/MS Drug Screening System version 3 (Shimadzu Corporation) [14]. Carbon monoxide hemoglobin saturation was determined using an AVOXimeter® 4000 (ITC, Edison, NJ, USA). Analysis of volatile organic compounds in blood was performed by headspace GC–MS at Foundation for Promotion of Material Science and Technology of Japan (Tokyo, Japan).

### LC–MS/MS conditions for quantification

Quantitative analyses were performed using a Nexera LC system coupled with an LCMS-8030 triple quadrupole mass spectrometer (Shimadzu Corporation). Chromatographic separation was achieved using an XB-C18 column (100 × 2.1 mm, particle size 2.6 µm; Phenomenex, Torrance, CA, USA) maintained at 40 °C. The mobile phase consisted of 10 mM of ammonium formate with 0.1% formic acid in water (A), and 10 mM ammonium formate with 0.1% formic acid in methanol (B). The flow rate was maintained at 0.4 mL/min. The gradient program was as follows: 5–95% B from 0 to 6 min, and 95% B until 7.5 min. At 7.6 min, the concentration of B was returned to 5% and maintained until 10 min. The MS was operated in the positive mode with an electrospray ionization interface. Ionization source conditions were as follows: nebulizer gas flow rate, 1.5 L/min; drying gas flow rate, 10 L/min; desolvation line temperature, 250 °C; and heat block temperature, 400 °C. High-purity argon and nitrogen were used as collision and nebulizer gases, respectively. Analytes were detected using multiple reaction monitoring (MRM) mode. The two product ions (*m/z*), a quantifier and a qualifier, were monitored for each analyte in MRM transitions (supplementary material Table S1). LabSolutions LCMS Ver. 5.109 software (Shimadzu Corporation) was used for data analysis.

### Extraction procedure for LDX in all samples and bromazepam in urine

Two hundred microliters of each postmortem sample, 100 μL of diluted aqueous ammonia solution (100-fold dilution of 28% ammonium solution), and 4 ng of phentermine (IS for LDX) or 50 ng of bromazepam-*d*_4_ (IS for bromazepam) were placed in a 1.5-mL tube and vortexed briefly. Next, 300 μL of acetonitrile was added to the QuEChERS Kit (2 mL tube containing magnesium sulfate and sodium acetate) [15], and the sample mixture was transferred into it. The tube was vortexed for 1 min and centrifuged at 8000 × *g* and 4 °C for 10 min. Subsequently, 2 μL of the supernatant and 30 μL of mobile phase A were co-injected into the LC–MS/MS.

### Extraction procedure for amphetamine, bromazepam, venlafaxine, and *O*-desmethylvenlafaxine in whole blood and gastric content samples

A hundred microliters of each postmortem sample, 200 μL of water, and 10 μL of the IS mixture were placed in a 1.5-mL tube and vortexed briefly. The following procedure was the same as for LDX.

### Extraction procedure for amphetamine, venlafaxine, and *O*-desmethylvenlafaxine in urine

The urine sample was diluted 20-fold with water, and the following hydrolysis was performed before extraction: 100 μL of the diluted urine, 10 μL of 1 M acetic acid/sodium acetate buffer (pH 5.0), 5 μL of β-glucuronidase, and 10 μL of the IS mixture were placed in a 1.5 mL tube and incubated at 50 °C for 2 h. After hydrolysis, 185 μL of water was added to the tube. The following procedure was the same as for LDX.

### Quantification methods

Amphetamine, bromazepam, venlafaxine, and *O*-desmethylvenlafaxine were quantified using the conventional matrix-matched calibration method (MMCM) [16] except for the gastric content sample, which were quantified using the standard addition method (SAM), because a similar blank matrix could not be prepared for the gastric contents [17]. Calibration curves of MMCM and SAM were generated with five and four calibration points, respectively. An appropriate IS for LDX could not be prepared; thus, LDX concentrations were determined using SAM, which compensated for recoveries and matrix effects.

### Method validations

In SAM for LDX in urine and gastric content samples, linearity (*R*^2^) and repeatability were examined [17]. Calibration curves (four calibration points) for SAM (see Table S2) were generated by repeating each point of calibrators 5 times and 5 quantitative values were obtained. The intraday repeatability of LDX was evaluated using the coefficient of variation (%CV) of the quantitative values in SAM. On the other hand, selectivity, linearity (*R*^2^), accuracy (%bias), and precision (%CV) were evaluated in MMCM for amphetamine in whole blood and urine, and for bromazepam, venlafaxine, and *O-*desmethylvenlafaxine in whole blood. Selectivity in MMCM was confirmed by demonstrating no signal interfering with the analytes and ISs in six different lots of human blank whole blood or urine. Intraday (*n* = 5) and interday (*n* = 15, 3 days repetition of intraday) accuracies and precisions in MMCM were determined by measuring quality control (QC) samples at the low and high points of their calibration curves. The limits of detection (LODs) of LDX in whole blood and for amphetamine in whole blood and urine were estimated by determining lower concentrations of QC sample that could be detected with S/N > 3. The QC samples for the LOD were prepared using three different lots of blank blood and urine. The LODs of LDX in urine and gastric contents were calculated using the equation in SAM proposed by Hasegawa et al. [17].

The stabilities of LDX in whole blood and urine were followed in vitro at ambient temperature, 4 °C, and − 20 °C. Nine microliters of 100,000 ng/mL LDX acetonitrile solution was placed into centrifuge tubes and the solvent (acetonitrile) was evaporated using a nitrogen stream. Eighteen milliliters of blank whole blood or urine was added to the tubes and mixed well, after which the samples were analyzed (0 h). Aliquots of the whole blood and urine were transferred into cryogenic vials and stored at ambient temperature (approximately 25 °C), 4 °C, and − 20 °C. Three different lots of blank whole blood and urine were used for each temperature. The whole blood samples stored at ambient temperature were analyzed after 6, 24, 48, 72, 96, 120, and 168 h. The blood samples stored at 4 °C were analyzed after 24, 48, 72, 96, 120, 144, and 168 h. The blood samples stored at − 20 °C and the urine samples at all temperatures were analyzed after 48, 96, and 168 h. The extraction procedure was the same as that described previously for LDX. Each analysis was repeated four times.

### Optical isomer separation for amphetamine

The identification of whether the amphetamines in the samples were *d*- or *l*-isomer was performed under the conditions as follows. Optical isomer separation was achieved using an Astec CHIROBIOTIC™ V2 column (250 × 2.1 mm, particle size 5 µm; Supelco^®^, Bellefonte, PA, USA) at ambient temperature (approximately 25 °C). Isocratic elution was used with 0.01% formic acid and 0.02% NH_4_OH in methanol as the mobile phase. The flow rate was maintained at 0.2 mL/min for 18 min. The injection volume was 1 µL. Other LC–MS/MS conditions were the same as described before.

## Results

### Basic toxicological results

The ethanol concentrations in femoral vein blood and urine were both < 0.1 mg/mL, and cyanide ion concentration in femoral vein blood was 130 ng/mL. Carbon monoxide hemoglobin saturation was 5.3% in left heart blood and 6.0% in right heart blood. n-Heptane concentration in cardiac whole blood was 410 ng/mL, and other volatile organic compounds such as benzene, toluene, and xylene were also detected at 1.00–100 ng/mL. The DRIVEN-FLOW^®^ M8-Z immunochemical screening of urine sample was positive for benzodiazepines. Alprazolam, amphetamine, atomoxetine, bromazepam, caffeine, sulpiride, and venlafaxine were detected in cardiac whole blood and urine samples using LC–MS/MS Drug Screening System given in the “Basic toxicological analyses” section. Then, quantitative analyses of amphetamine, bromazepam, venlafaxine, and *O*-desmethylvenlafaxine were performed based on the semiquantitative values obtained in the drug screening test. When the LDX reference standard data were compared with those of the urine and gastric content extracts, the three product ions and retention times were identical, and thus, LDX was determined to be detected.

**Fig. 2:**
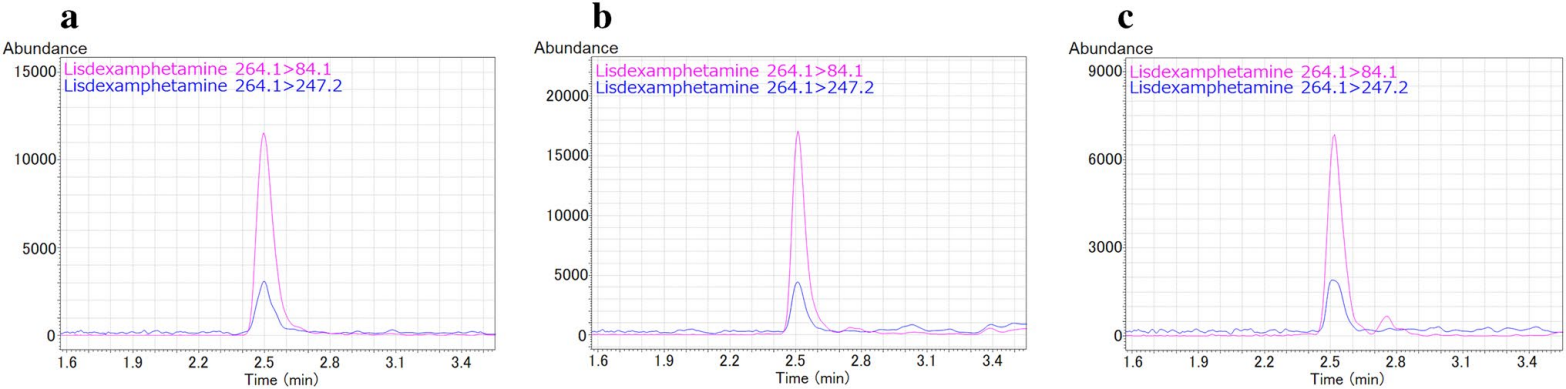
Multiple reaction monitoring (MRM) chromatograms for LDX. **a** LDX standard solution (2 ng in 200 µL acetonitrile); **b** urine extract; **c** gastric contents extract

Table 1 shows concentrations of LDX, amphetamine, bromazepam, venlafaxine, and *O-*desmethylvenlafaxine in each sample. The MRM chromatograms for LDX are shown in Fig. 2. Abundance ratio of the urine extract (Fig. 2b) to the gastric contents extract (Fig. 2c) for LDX was approximately double; however, the concentration ratio was approximately 7 (Table 1), indicating that the sample matrices had strong effects on the extraction recovery and/or matrix effect for LDX. Except for LDX in urine and gastric contents, amphetamine in whole blood and urine, and bromazepam, venlafaxine, and *O-*desmethylvenlafaxine in whole blood, the drug concentrations have not been validated (Table 1); the values without validation are considered to be tentative quantitative ones.

**Table 1:** Concentrations (ng/mL) of lisdexamfetamine (LDX), amphetamine, bromazepam, venlafaxine, and *O-*desmethylvenlafaxine in each sample

	Femoral blood	Heart blood	Urine	Gastric contents
LDX	*nd*	*nd*	30.9	4.42
Amphetamine	329	510	2970	915^a^
Bromazepam	460	480	235^a^	10,100^a^
Venlafaxine	654	873	3920^a^	1090^a^
*O*-Desmethylvenlafaxine	630	901	30,200^a^	930^a^

*nd* not detected

^a^Not validated

### Validation data

No interfering signals were observed in the retention times of all analytes and ISs from six different lots of human blank whole blood or urine in MMCM. The calibration equations and coefficients of determination (*R*^2^) for each analyte in each sample are shown in Table S2. The means and standard deviations of the LDX concentrations in urine and gastric contents obtained from intraday five experiments and repeatability by the SAM are shown in Table S3. The accuracy and precision data for the quantification of amphetamine in whole blood and urine by MMCM are shown in Table S4; those for the quantification of bromazepam, venlafaxine, and *O*-desmethylvenlafaxine in whole blood are shown in Table S5. The LODs of LDX and amphetamine in blood, urine, and gastric contents are shown in Table S6.

**Fig. 3:**
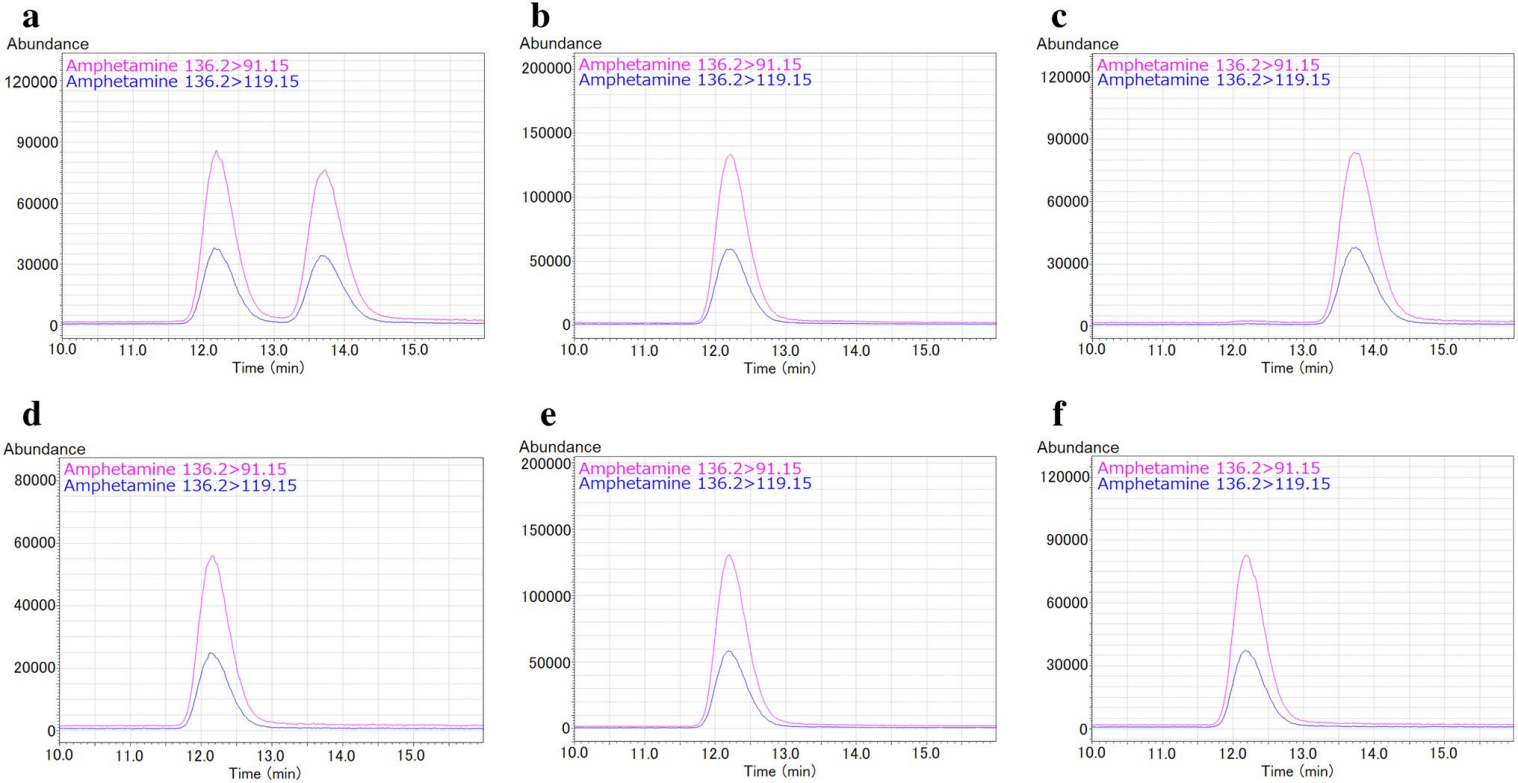
MRM chromatograms for *d*-amphetamine and *l*-amphetamine. **a** *d*-Amphetamine/*l*-amphetamine reference standard solution (20 ng in 200 μL acetonitrile); **b** *d*-amphetamine reference standard solution; **c** *l*-amphetamine reference standard solution; **d** heart whole blood extract; **e** urine extract; **f** gastric contents extract

LC–MS/MS chromatograms for *d*-amphetamine and *l*-amphetamine are shown in Fig. 3. The amphetamine isomer detected in the samples of this case was identified to be *d*-amphetamine by optical isomer separation.

**Fig. 4:**
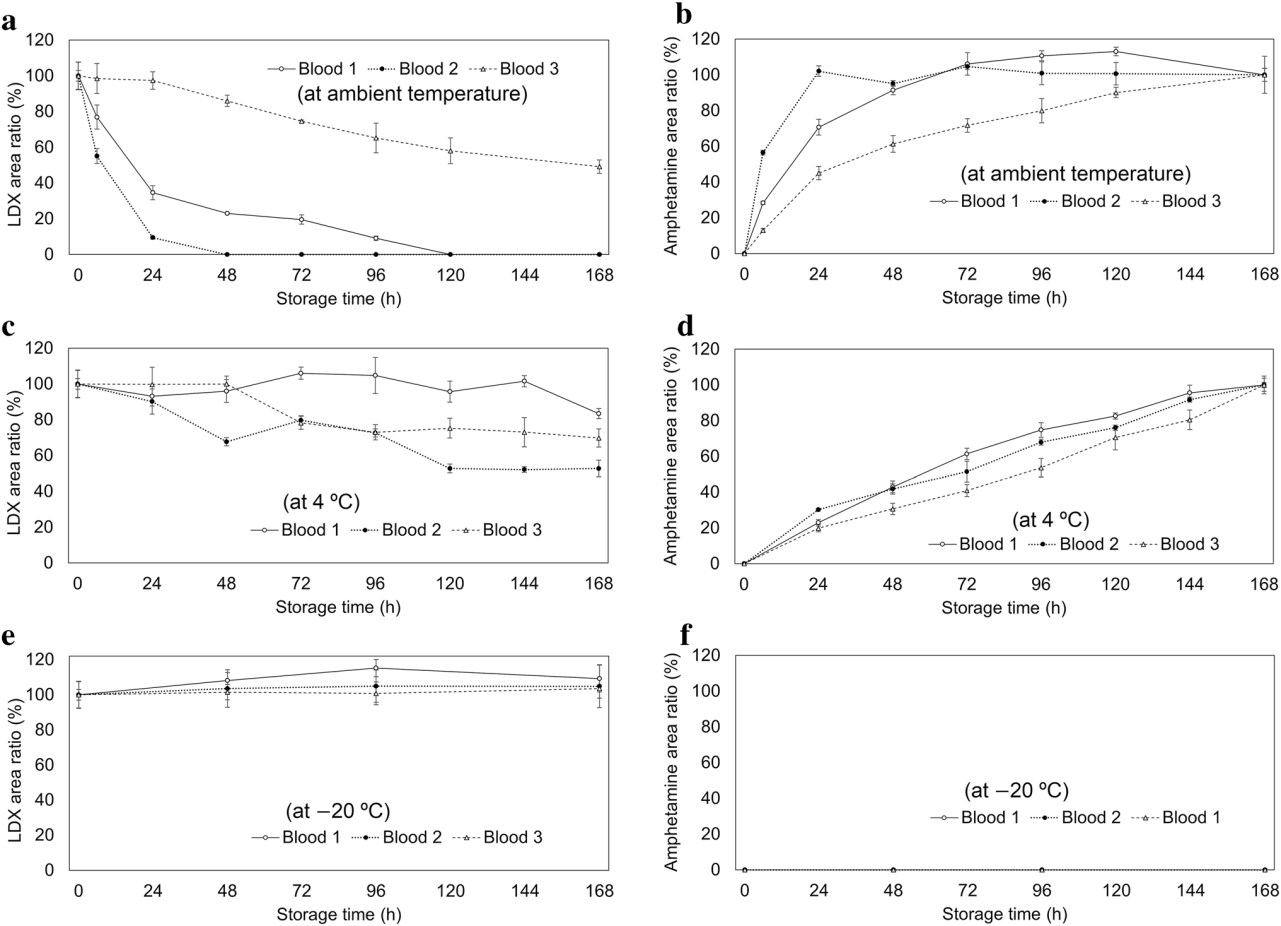
In vitro stabilities of LDX in whole blood [mean ± standard deviation (SD), *n* = 4]. **a** LDX/internal standard (IS) area ratio when stored at ambient temperature (approximately 25 °C); **b** amphetamine/IS area ratio when stored at ambient temperature; **c** LDX/IS area ratio when stored at 4 °C; **d** amphetamine/IS area ratio when stored at 4 °C; **e** LDX/IS area ratio when stored at − 20 °C; **f** amphetamine/IS area ratio when stored at − 20 °C

**Fig. 5:**
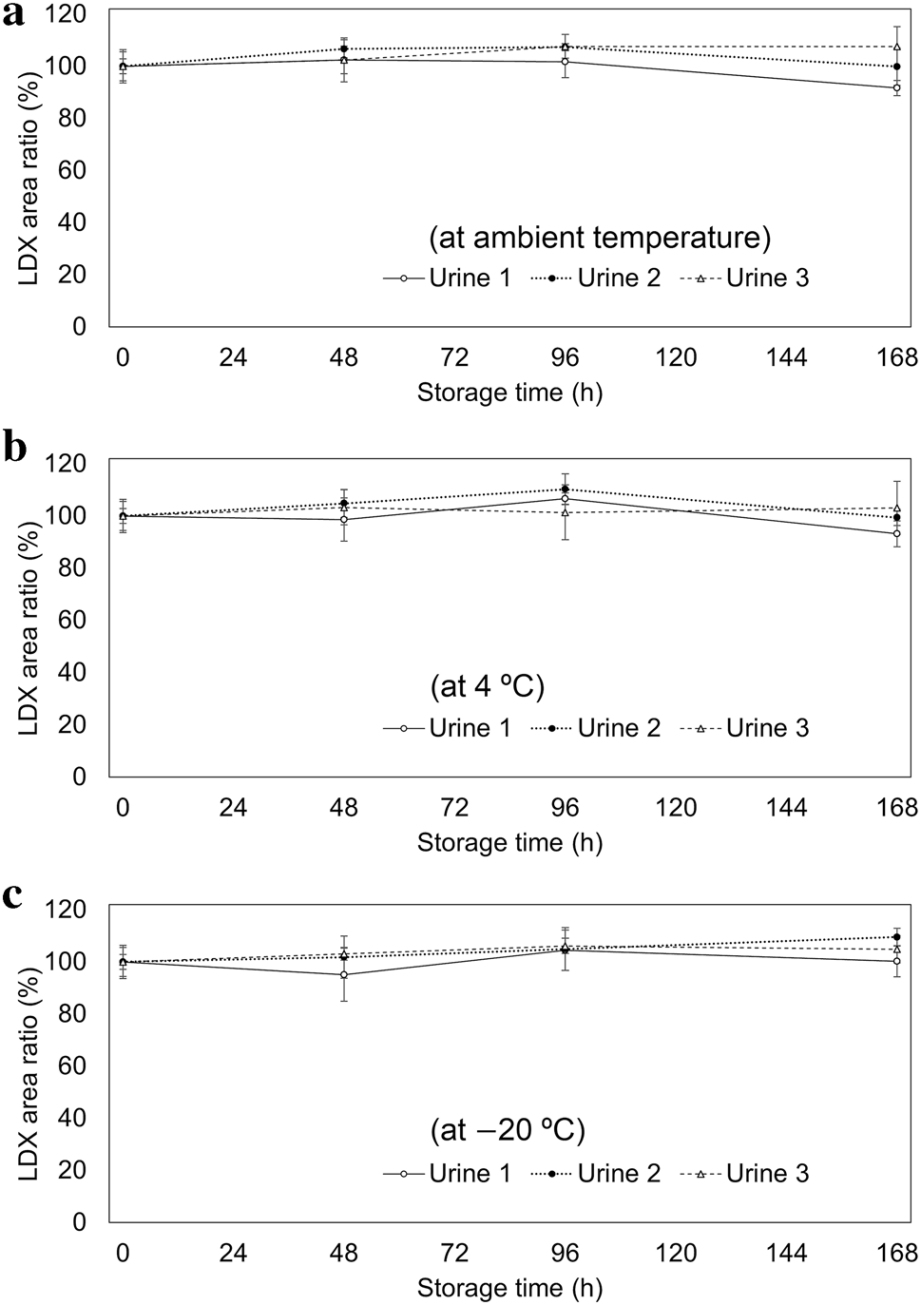
In vitro **s**tabilities of LDX in urine (mean ± SD, *n* = 4). **a** LDX/IS area ratio when stored at ambient temperature; **b** LDX/IS area ratio when stored at 4 °C; **c** LDX/IS area ratio when stored at − 20 °C

Figures 4 and 5 show the in vitro stability study results with whole blood and urine, respectively. For LDX, the mean value of the LDX/IS peak area ratio of each sample before storage (0 h) was 100%; for amphetamine, the mean value of the amphetamine/IS peak area ratio of each sample analyzed after 168 h storage was 100%. The LDX in whole blood was unstable when stored at ambient temperature; after 48 h of storage, LDX was not detected in one sample. The amphetamine in whole blood increased from 0 to more than 100% after 24 h in the same sample (for blood 2). When stored at 4 °C, the LDX in whole blood was reduced to half of its maximum after 168 h (for blood 2) and was more stable than that at ambient temperature; the amphetamine in whole blood gradually increased for 168 h. The LDX in whole blood did not decrease and the amphetamine did not increase when stored at − 20 °C for 168 h. The LDX in urine was stable (within ± 15%) at each temperature for 168 h.

### Discussion

In Japan, more than 73% of illegal drug abuse is attributed to methamphetamine [18]; methamphetamine is usually detected together with amphetamine. However, in the present case, only amphetamine was detected. Forty days after the autopsy, the police conducted a hospital investigation and found that the deceased had been prescribed Vyvanse®, which uncovered the possibility that he had been taking LDX. Then, LDX was detected in urine and gastric contents, which were kept frozen in a freezer. Although the concentrations of LDX in whole blood were below the LOD, those in the urine and gastric content samples could be quantified (Table 1), showing that LDX could be detected even in postmortem samples. Linearities for LDX, amphetamine, bromazepam, venlafaxine, and *O-*desmethylvenlafaxine were considered good because the *R*^2^ values were greater than 0.99 for all calibration curves (Table S2). In addition, both precisions and repeatabilities in the present case were less than 15% (Tables S3–S5), which met the U.S. Food and Drug Administration guidance criteria [19]. Thus, the present method using LC–MS/MS may be suitable for accurate quantification of these compounds. Because the elimination half-life of LDX in living humans is generally less than 1 h [20], the near-total elimination of LDX from the blood before death of the present victim could be reasonably assumed. Furthermore, the in vitro stability study indicated that hydrolysis of LDX in whole blood could have also possibly occurred even after death at ambient temperature (Fig. 4). Similarly, Sørensen et al. [21] reported that when fluoride-untreated blood samples with LDX were stored at 20 °C for 24 h, less than half of the initial concentration was recovered. These results indicate that blood samples should be collected to be frozen as soon as possible at autopsy to ensure the detectability of LDX upon its analyses. However, the in vitro stability study revealed that LDX in urine was not hydrolyzed within 7 days even at ambient temperature (Fig. 5). These results suggest that urine samples may be most useful for detecting LDX during the postmortem period.

Amphetamine is a molecule with a chiral center and generally exists as a racemate with two isomers, *d*-amphetamine and *l*-amphetamine [9]. On the other hand, no residual impurities of *l*-amphetamine exist in the LDX formulation [9], and no racemization occurs during metabolism [22, 23]. In this case, LDX and *d*-amphetamine were detected, whereas methamphetamine and *l*-amphetamine were not detected (Figs. 2, 3). Therefore, the amphetamine in the present case was likely produced from the hydrolysis of LDX. In other words, the toxicological analysis appropriately indicated that no illicit drugs had been used. Furthermore, selegiline, which is used for reducing symptoms in early-stage Parkinson’s disease, is metabolized to desmethylselegiline, *l*-methamphetamine, and *l*-amphetamine [24]. Thus, optical isomer separation testing is necessary to determine whether amphetamine detected is a medical or illicit drug.

The LODs of methamphetamine and amphetamine for DRIVEN-FLOW® M8-Z are 500 and 85,000 ng/mL, respectively. Although there are many different types of immunoassay kits, a simple test using a urine sample may miss the presence of amphetamine because of its low sensitivity for amphetamine. Therefore, a drug toxicology screening test using analytical instruments should be routinely performed at autopsy.

Symptoms reported after LDX exposure are similar to those of amphetamine toxicity such as increased blood pressure, tachycardia, agitation, and vomiting [25]. Clinical manifestations of stimulant toxicity include effects on the nervous and cardiac systems, and secondary complications such as effects on the kidney, lung, and digestive systems [26]. Krishnan and Montcrief [27] reported that rats died after oral administration of high doses of LDX. Although successful resuscitation after LDX overdose has been reported [28, 29], deaths from LDX intoxication will take place in the future. In the present case, none of the drugs in femoral vein blood did not reach fatal concentrations [30]; the cause of death was obviously thermal burns by fire.

## Conclusions

In this case report, we encountered a forensic autopsy case, in which amphetamine was detected in qualitative analysis, and then quantitative analyses of LDX were performed using LC–MS/MS. The concentrations of LDX were 30.9 and 4.42 ng/mL in urine and gastric content samples, respectively. In the whole blood samples, LDX could not be detected. The amphetamine isomer detected in the samples of this case was identified to be only *d*-amphetamine derived from metabolism of LDX by optical isomer separation testing. The results in the present case may be useful in interpreting whether or not the amphetamine detected in cadavers is a metabolite of LDX. To our knowledge, this is the first report to demonstrate LDX and *d*-amphetamine from samples in an autopsy case.

## Supplementary Information

Below is the link to the electronic supplementary material.

Supplementary file1 (DOCX 31 KB)
