# Supplementary material for: Detection of lisdexamfetamine and its metabolite d-amphetamine in urine and gastric contents collected from a cadaver at forensic autopsy
Source: Forensic Toxicol. 2022 Dec 23;41(2):309–17. doi: 10.1007/s11419-022-00654-6 (PMC10310599; doi:10.1007/s11419-022-00654-6)
Supplement: Supplementary file 1 — Supplementary file1 (DOCX 31 KB) [file 11419_2022_654_MOESM1_ESM.docx]

**Supplementary material**

Detection of lisdexamfetamine and its metabolite d-amphetamine in urine and gastric contents collected from a cadaver at forensic autopsy

Suguru Torimitsu^1,2^・Kanju Saka^1^・Kanako Noritake^1^・Akira Namera^3^・Yohsuke Makino^1,2^・Rutsuko Yamaguchi^1,2^・Hirotaro Iwase^1,2^

^1^ Department of Forensic Medicine, Graduate School of Medicine, The University of Tokyo, 7-3-1 Hongo, Bunkyo-ku, Tokyo, 113-0033, Japan

^2^ Department of Legal Medicine, Graduate School of Medicine, Chiba University, 1-8-1 Inohana, Chuo-ku, Chiba-shi, Chiba, 260-8670, Japan

^3^ Department of Forensic Medicine, Graduate School of Biomedical and Health Sciences, Hiroshima University, Kasumi 1-2-3, Minami-ku, Hiroshima, 734-8553, Japan

Corresponding Author

Suguru Torimitsu (🖂)

Tel: +81-3-5841-3367

Fax: +81-3-5841-3366

E-mail: torimitsu@m.u-tokyo.ac.jp

**Table S1** Retention times, precursor ions, product ions, and collision energies for analytes

| Analyte | Retention time (min) | Precursor ion (*m*/*z*) | Product ion (*m*/*z*) | Collision energy (V) |
| --- | --- | --- | --- | --- |
| Lisdexamfetamine (LDX) | 2.57 | 264.1 | 84.1^a^ | 26 |
|  |  | 264.1 | 247.2 | 16 |
| Amphetamine | 2.84 | 136.2 | 91.1 ^a^ | 20 |
|  |  | 136.2 | 119.1 | 16 |
| Phentermine (IS)^b^ | 3.35 | 150.0 | 91.0 ^a^ | 23 |
|  |  | 150.0 | 133.0 | 17 |
| *O*-Desmethylvenlafaxine | 3.56 | 264.2 | 58.0 ^a^ | 22 |
|  |  | 264.2 | 246.0 | 13 |
| Venlafaxine-*d*_6_ (IS)^c^ | 4.43 | 284.3 | 58.1 ^a^ | 22 |
|  |  | 284.3 | 266.1 | 14 |
| Venlafaxine | 4.44 | 278.3 | 58.1 ^a^ | 22 |
|  |  | 278.3 | 260.1 | 14 |
| Bromazepam-*d*_4_ (IS)^d^ | 4.98 | 320.0 | 186.1 ^a^ | 35 |
|  |  | 320.0 | 213.1 | 30 |
| Bromazepam | 4.99 | 316.1 | 182.0 ^a^ | 37 |
|  |  | 316.1 | 209.1 | 31 |

*IS* internal standard

^a^ Quantifier ion

^b^ IS for LDX and amphetamine

^c^ IS for *O*-desmethylvenlafaxine and venlafaxine

^d^ IS for bromazepam

**Table S2** Calibration equations and coefficients of determination (*R*^2^) for each analyte in each sample by matrix-matched calibration method (MMCM) (A) or standard addition method (SAM) (B)

|  | Method | Conc. added (ng/mL) | Range (ng/mL) | Equation | *R*^2^ |
| --- | --- | --- | --- | --- | --- |
| Whole blood |  |  |  |  |  |
| Amphetamine | A |  | 50–1000 | *y* = 0.00182 *x* − 0.0275 | 0.998 |
| Bromazepam | A |  | 100–2000 | *y* = 0.00146 *x* − 0.0218 | 0.999 |
| Venlafaxine | A |  | 100–2000 | *y* = 0.00259 *x* − 0.0361 | 1.000 |
| *O*-Desmethylvenlafaxine | A |  | 100–2000 | *y* = 0.00240 *x* − 0.0612 | 1.000 |
| Urine |  |  |  |  |  |
| LDX | B | 0, 20, 40, 80 |  | *y* = 0.00618 *x* + 0.191 | 0.999 |
| Amphetamine | A |  | 1000–20,000 | *y* = 0.0000854 *x* − 0.00667 | 0.998 |
| Bromazepam | A |  | 50–1000 | *y* = 0.00280 *x* − 0.0113 | 1.000 |
| Venlafaxine | A |  | 2000–40,000 | *y* = 0.000125 *x* − 0.00445 | 1.000 |
| *O*-Desmethylvenlafaxine | A |  | 2000–40,000 | *y* = 0.000123 *x* − 0.0515 | 1.000 |
| Gastric contents |  |  |  |  |  |
| LDX | B | 0, 2.5, 5.0, 10 |  | *y* = 0.0243 *x* + 0.110 | 0.999 |
| Amphetamine | B | 0, 500, 1000, 2000 |  | *y* = 0.00189 *x* + 1.73 | 1.000 |
| Bromazepam | B | 0, 5000, 10,000, 20,000 |  | *y* = 0.00148 *x* + 15.0 | 0.998 |
| Venlafaxine | B | 0, 500, 1000, 2000 |  | *y* = 0.00335 *x* + 3.65 | 0.997 |
| *O*-Desmethylvenlafaxine | B | 0, 500, 1000, 2000 |  | *y* = 0.00206 *x* + 1.92 | 0.998 |

*y* peak area of analyte/peak area of IS

*x* concentration of analyte

**Table S3** Means and standard deviations of LDX concentrations in urine and gastric content samples obtained from five intraday experiments and repeatability obtained by SAM

|  | Conc. (ng/mL) | Intraday repeatability (%CV) |
| --- | --- | --- |
| Urine | 30.9 ± 2.4 | 7.66 |
| Gastric contents | 4.42 ± 0.42 | 9.47 |

*%CV* percent coefficient of variation

**Table S4** Accuracy and precision data for quantitation of amphetamine in whole blood and urine obtained by MMCM

|  | Conc. (ng/mL) | Intraday (*n* = 5) | |  | Interday (*n* = 15) | |
| --- | --- | --- | --- | --- | --- | --- |
|  |  | Accuracy (%bias) | Precision (%CV) |  | Accuracy (%bias) | Precision (%CV) |
| Blood | 100 | −2.74 | 3.46 |  | 3.50 | 5.90 |
|  | 1000 | −9.12 | 1.64 |  | 1.30 | 8.13 |
| Urine | 2000 | 8.58 | 3.01 |  | 6.73 | 4.09 |
|  | 20,000 | 0.66 | 4.02 |  | −0.84 | 5.17 |

*%CV* percent coefficient of variation

**Table S5** Accuracy and precision data for quantitation of bromazepam, venlafaxine, and *O-*desmethylvenlafaxine in whole blood obtained by MMCM

|  | Conc. (ng/mL) | Intraday (*n* = 5) | |  | Interday (*n* = 15) | |
| --- | --- | --- | --- | --- | --- | --- |
|  |  | Accuracy (%bias) | Precision (%CV) |  | Accuracy (%bias) | Precision (%CV) |
| Bromazepam | 200 | 3.85 | 3.66 |  | 3.09 | 4.07 |
|  | 2000 | −2.83 | 3.50 |  | −4.22 | 3.29 |
| Venlafaxine | 200 | 0.52 | 1.14 |  | 5.09 | 2.24 |
|  | 2000 | −3.21 | 0.47 |  | 1.90 | 2.13 |
| *O*-Desmethylvenlafaxine | 200 | −4.00 | 2.51 |  | −4.51 | 1.73 |
|  | 2000 | 2.83 | 1.45 |  | −0.90 | 1.46 |

**Table S6** Limits of detection (ng/mL) of LDX and amphetamine in whole blood, urine, and gastric contents

|  | Blood | Urine | Gastric contents |
| --- | --- | --- | --- |
| LDX | 4.00^a^ | 0.676^b^ | 0.173^b^ |
| Amphetamine | 5.00^a^ | 100^a^ | 15.8^b^ |

^a^ Experimental value detected with S/N > 3

^b^ Calculated value obtained from the equation described in ref. [17]
